# The pharmacological properties and corresponding mechanisms of farrerol: a comprehensive review

**DOI:** 10.1080/13880209.2021.2006723

**Published:** 2021-11-30

**Authors:** Xiaojiang Qin, Xinrong Xu, Xiaomin Hou, Ruifeng Liang, Liangjing Chen, Yuxuan Hao, Anqi Gao, Xufeng Du, Liangyuan Zhao, Yiwei Shi, Qingshan Li

**Affiliations:** a School of Public Health, Shanxi Medical University, Taiyuan, Shanxi, China; b Department of Pharmacology, Shanxi Medical University, Shanxi, China; c Department of Exercise Rehabilitation, Shanxi Medical University, Taiyuan, Shanxi, China; d Department of Respiratory and Critical Care Medicine, Shanxi Medical University Affiliated First Hospital, Taiyuan, Shanxi, China; e School of Pharmaceutical Science, Shanxi Medical University, Taiyuan, Shanxi, China; f Shanxi Key Laboratory of Chronic Inflammatory Targeted Drugs, School of Materia Medica, Shanxi University of Traditional Chinese Medicine, Taiyuan, Shanxi, China

**Keywords:** Anti-inflammatory, antioxidant, vasoactive, antitumor, antimicrobial, molecular mechanisms

## Abstract

**Context:**

Farrerol, a typical natural flavanone isolated from the traditional Chinese herb ‘Man-shan-hong’ [*Rhododendron dauricum* L. (Ericaceae)] with phlegm-reducing and cough-relieving properties, is widely used in China for treating bronchitis and asthma.

**Objective:**

To present the anti-inflammatory, antioxidant, vasoactive, antitumor, and antimicrobial effects of farrerol and its underlying molecular mechanisms.

**Methods:**

The literature was reviewed by searching PubMed, Medline, Web of Knowledge, Scopus, and Google Scholar databases between 2011 and May 2021. The following key words were used: ‘farrerol,’ ‘flavanone,’ ‘anti-inflammatory,’ ‘antioxidant,’ ‘vasoactive,’ ‘antitumor,’ ‘antimicrobial,’ and ‘molecular mechanisms’.

**Results:**

Farrerol showed anti-inflammatory effects mainly mediated via the inhibition of interleukin (IL)-6/8, IL-1β, tumour necrosis factor(TNF)-α, NF-κB, NO, COX-2, JNK1/2, AKT, PI3K, ERK1/2, p38, Keap-1, and TGF-1β. Farrerol exhibited antioxidant effects by decreasing JNK, MDA, ROS, NOX4, Bax/Bcl-2, caspase-3, p-p38 MAPK, and GSK-3β levels and enhancing Nrf2, GSH, SOD, GSH-Px, HO-1, NQO1, and p-ERK levels. The vasoactive effects of farrerol were also shown by the reduced α-SMA, NAD(P)H, p-ERK, p-Akt, mTOR, Jak2, Stat3, Bcl-2, and p38 levels, but increased OPN, occludin, ZO-1, eNOS, CaM, IP3R, and PLC levels. The antitumor effects of farrerol were evident from the reduced Bcl-2, Slug, Zeb-1, and vimentin levels but increased p27, ERK1/2, p38, caspase-9, Bax, and E-cadherin levels. Farrerol reduced α-toxin levels and increased NO production and NF-κB activity to impart antibacterial activity.

**Conclusions:**

This review article provides a theoretical basis for further studies on farrerol, with a view to develop and utilise farrerol for treating of vascular-related diseases in the future.

## Introduction

Flavanone, a common class of polyphenol compounds naturally present in fruits, vegetables, nuts, seeds, flowers, and bark, exhibits a wide range of pharmacological properties, including antioxidant, anti-inflammatory, vasodilatory, antitumor, and antibacterial effects (Zhu et al. 2007; Zhao J et al. 2012; Abotaleb et al. 2018; Chen et al. 2019; Farhadi et al. 2019). A common flavanone, farrerol, that is isolated from the traditional Chinese herb ‘Man-shan-hong’ [the dried leaves of *Rhododendron dauricum* L. (Ericaceae)] has phlegm-reducing and cough-relieving properties, and is thus widely used in China for treating bronchitis and asthma (Li et al. 2014; Liu et al. 2016). However, to overcome its poor extraction yield on extraction from natural resources, farrerol and its derivatives have been successfully synthesised using multiple chemical methods to investigate their novel pharmacological properties (Shi et al. 2010, 2011; Zhang et al. 2019). Consequently, research on farrerol in the field of medicine has progressed rapidly in recent years. Moreover, farrerol enhances in-frame integration of exogenous donor DNA and has the ability to efficiently generate knock-in mice with germline transmission capacity (Zhang, Murugesan, et al. 2020). Consequently, research on farrerol in the field of medicine has progressed rapidly in recent years with many novel molecular mechanisms having been characterized.

Many studies on farrerol have investigated its biological activities; however, the different pharmacological activities of farrerol and its associated molecular mechanisms remain unclear. Therefore, we have systematically reviewed the pharmacological properties and underlying mechanisms of farrerol and have identified challenges, hoping to provide directions and ideas for future research.

## Methods

Medline/PubMed, Web of Knowledge, Scopus, and Google Scholar were searched to find studies on the anti-inflammatory, antioxidant, vasoactive, antitumor, and antibacterial effects of farrerol, published from 2011 until the end of May 2021. The following key words were used: ‘farrerol,’ ‘flavanone,’ ‘anti-inflammatory,’ ‘antioxidant,’ ‘vasoactive,’ ‘antitumor,’ ‘antimicrobial,’ and ‘molecular mechanisms’.

## Anti-inflammatory effect of farrerol

Inflammation plays a vital role in the body's defense response, limiting inflammatory cytokines and facilitating the repair of damaged parts of the body (Ray and Rai 2017; Luscher 2019). In contrast, excessive inflammatory response can cause degeneration and necrosis of the cells and tissues (Afonina et al. 2017; Kearney & Martin 2017; Abplanalp et al. 2020). For a long time, steroids and cyclooxygenase inhibitors have been used to treat diseases associated with inflammatory response, but their relative limitations have made developing new replacement drugs a priority (Ci et al. 2012; Zarrin et al. 2021).

Flavonoids are a large group of polyphenolic natural products that are widely distributed in higher plants and are well known to have a variety of therapeutic activities (Deng et al. 2021; Zhan et al. 2021). Some flavonoids isolated from Rhododendron roots have potential anti-inflammatory agents, based on the results of dose-dependent inhibition of the expressions of inflammatory mediators (Mulvihill et al. 2016; Rengasamy et al. 2019; Zhang et al. 2021). Farrerol, a flavonoid extracted initially from Rhododendron, is a traditional Chinese herbal medicine (Zhao et al. 2010; Fu et al. 2012). Although the anti-inflammatory mechanism of farrerol has not been clearly elucidated so far, it has therapeutic advantages in inflammatory diseases.

*In vivo* experiments, the anti-inflammatory activity of farrerol was reported by Xin Ran (2018) and Xiong (2013). Ci et al. (2012) found that farrerol markedly alleviated the allergic airway inflammation in an allergic asthma model, and its mechanism of action was related to the activation of phosphorylation of Akt and nuclear factor (NF-κB) subunit p65. Ci et al. (2012) proved that farrerol could exert anti-inflammatory effects in the treatment of asthma by inhibiting the PI3K and NF-κB signalling pathways. Ci et al. (2012) found that farrerol significantly inhibited T cell-mediated delayed-type hypersensitivity in female BALB/c mice. The mechanism of action may be related to the downregulation of NF-κB activation and nuclear factor of activated T cell 2 signal transduction pathways (Taylor et al. 2013). Additionally, in 2018, Ran et al. (2018) reported that farrerol administration significantly improved the weight changes, clinical scores, colonic length and intestinal epithelial barrier damage and markedly decreased inflammatory cytokine production in TNBS-induced mice. This anti-inflammatory effect was mediated by decreasing the production of interlekin (IL)-1β, IL-6, and tumour necrotic factor (TNF)-α and increasing the expression of claudin-1, zonula occludens 1 (ZO-1), and occludin (Ran et al. 2018). Li et al. (2018) reported that farrerol could ameliorate pathological damage in the mammary glands; attenuate myeloperoxidase (MPO) activity; and inhibit the production of pro-inflammatory mediators and phosphorylation of AKT, NF-κB p65, p38, and ERK1/2 in lipopolysaccharide (LPS)-induced mouse mastitis.

**Figure 1.**
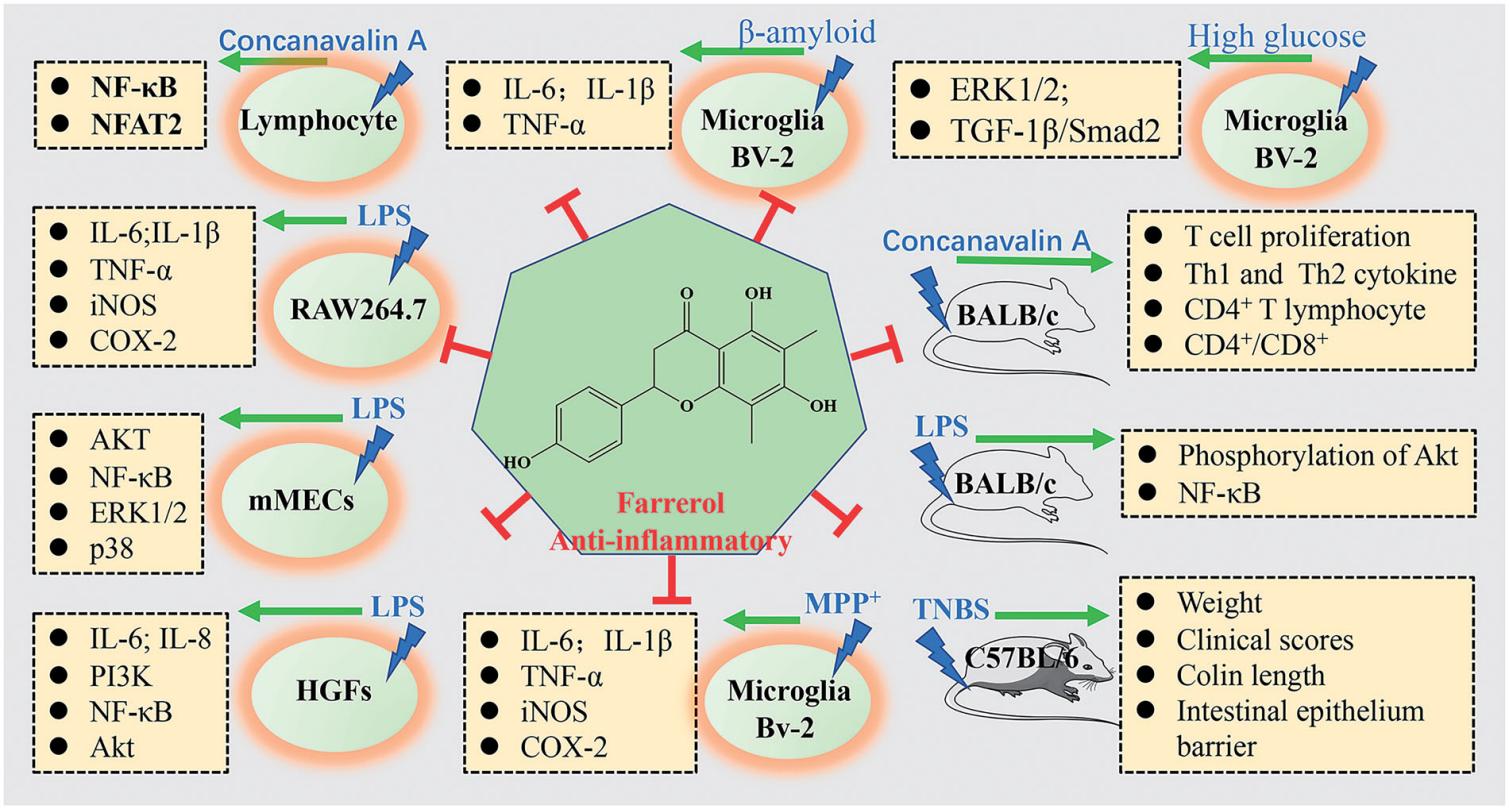
The anti-inflammatory mechanism of farrerol and related molecules.

*In vitro* experiments, Ran et al. (2018) also revealed that farrerol remarkably decreased the production of inflammatory mediators, including IL-1β, IL-6, and TNF-α, and the expression of COX-2 and iNOS in LPS-induced RAW264.7 cells by suppressing AKT, ERK1/2, JNK1/2, and NF-κB p65 phosphorylation. Similarly, Li et al. (2018) confirmed that farrerol could inhibited LPS-induced inflammatory response and the related signalling pathways in mouse mammary epithelial cells (mMECs). Zhang et al. (2015) found that farrerol exhibited anti-inflammatory effects by preventing IL-6β-induced P13K/Akt phosphorylation and significantly inhibiting IL-6β-induced NO and PGE2 production and expression of iNOS and COX-2 in chondrocytes. Wang et al. (2016) demonstrated that farrerol suppressed LPS-induced IL-6 and IL-8 expression, both at the mRNA and protein levels. Farrerol significantly inhibited the phosphorylation of PI3K and AKT, thus attenuating IL-6 and IL-8 production and inhibiting NF-κB P65 phosphorylation and IκBα degradation in LPS-stimulated human gingival fibroblasts. Moreover, Cui, Guo, et al. (2019) found that farrerol attenuated Aβ-induced inflammation in BV-2 cells by enhancing the activation of the Nrf2/Keap1 pathway. In 2019, Cui et al. reported that farrerol could inhibit the TLR4 signalling pathway to alleviate MPP^+^-induced inflammatory response in BV-2 cells and suppress proinflammatory mediators’ production (mediators, such as iNOS, COX-2, IL-1β, IL-6, TNF-α, NO, and PGE2) in LPS-treated BV-2 cells. Farrerol inhibits NF-κB p65 and AKT phosphorylation, but it has no significant effect on MAPKs phosphorylation (ERK1/2, p38, and JNK1/2) (Cui, Guo, et al. 2019; Li Y et al. 2019). The results are shown in Table 1 and Figure 1.

**Table 1.** Anti-inflammatory and antioxidant activity of farrerol and its underlying signalling pathway mechanism.

Activity	Cell/animal	Mechanism	Signalling pathway	References
Anti-inflammatory	Microglia BV-2	↓IL-6; IL-1β; TNF-α;	TLR4	[Cui et al. 2019]
		iNOS; COX-2		
	Mesangial	↓ERK1/2; TGF-1β/Smad2	ROS/Nox4/ERK1/2	[Chen et al. 2019]
	mMECs	↓AKT; NF-κB; ERK1/2; p38	AKT/NF-κB p65; ERK1/2; p38	[Li et al. 2018]
	RAW264.7; C57BL/6	↓IL-6; IL-1β; TNF-α; COX-2; iNOS; AKT; ERK1/2; JNK1/2; NF-κB p65 phosphorylation	ERK1/2; JNK1/2;	[Ran et al. 2018]
			NF-κB	
	Lymphocyte; BALB/ c mice	↓NFAT2; NF-κB	NF-κB and NFAT2	[Xiong et al. 2013]
		**↑**IκB		
	HGFs	↓IL-6; IL-8; PI3K; Akt phosphorylation;	P13K/Akt/NF-κB	[Wang et al. 2016]
		NF-κB		
	Chondrocytes	↓NO; PGE2; COX-2; iNOS; NF-κB; p-P13K; p-Akt	P13K/Akt/NF-κB	[Zhang et al. 2015]
	BALB/c mice	↓p-Akt；NF-κB	/	[Ci et al. 2012]
Antioxidant	HepG2	↓JNK	AMPK/AKT	[Wang et al. 2016]
		**↑**Nrf2		
	EA.hy926	↓GSK-3β	Nrf2-ARE	[Yan et al. 2020]
	ARPE-19	↓ROS; MDA; PARP; Bax/Bcl-2; Caspase-8; Caspase-9	Akt; MAPK	[Ma et al. 2019]
		**↑**GSH; SOD; HO-1; NQO1; GCLM		
	RAW264.7	↓JNK	Nrf2 mediated HO-1	[Ci et al. 2015]
		**↑**HO-1; pAKT/AKT; p-p38/p38; p-ERK/ERK		
	EA.hy926	↓MDA; ROS; Bax; Caspase-3; p-p38 MAPK	/	[Li et al. 2013]
		**↑**SOD; GSH-Px; Bcl-2		
Anti-inflammatory/ antioxidant	MTECs; HK-2	↓Keap-1; NOX4	Oxidation; Inflammation	[Ma et al. 2019]
		**↑**HO-1; NQO1		
	Microglia BV-2	↓ROS; MDA; IL-6; IL-1β; TNF-α; Keap-1	Nrf2/Keap1	[Cui et al. 2019]
		**↑**SOD; Nrf2; HO-1; NQO1		

## Antioxidant effect

Oxidative stress is considered to be involved in the pathogenesis of various diseases and is defined as an imbalance between the production of free radicals and reactive metabolites (Takahashi et al. 2018; Hayes et al. 2020; Mendiola et al. 2020). Normal amounts of reactive oxygen species (ROS) can produce beneficial physiological effects, including liver detoxification and cell division regulation. However, surplus free radicals have adverse effects on the body, such as inducing DNA damage and affecting the DNA damage response (DDR) (Ardolino et al. 2011; Panikkanvalappil et al. 2013; Srinivas et al. 2019). Under oxidative stress, high levels of ROS contribute to several chronic human diseases, such as cardiovascular diseases and rheumatism (Blanco et al. 2018; Gong et al. 2018; Chen et al. 2019; Zhang Y et al. 2020).

Ci et al. published four articles between 2015 and 2020 on the antioxidant effects of farrerol, emphasising on the following: First, farrerol induced Hnti-oxidant O-1 protein expression in a time- and dose-dependent manner in RAW 264.7 macrophage cells, suggesting that its antioxidative property accounts for the induction of Heme Oxygenase-1 (HO-1) expression. In addition, farrerol attenuated the phosphorylation of c-Jun N-terminal kinase (JNK), extracellular signal-regulated kinase (ERK), and p38 mitogen-activated protein kinase (p38) and the activation of phosphorylated nuclear factor-κB (p-NF-κB) and nucleotide-binding domain (NOD)-like receptor protein 3 (NLRP3) (Ci et al. 2015). Second, farrerol could protect against acetaminophen-induced hepatotoxicity, which may be related to the activation of Nrf2 (Wang et al. 2019). Third, farrerol improved cisplatin-induced nephrotoxicity by ameliorating oxidative and activating nuclear factor erythroid 2-related factor 2 (Nrf2). Farrerol effectively activated Nrf2 and subsequently increased the expression of Nrf2-targeted antioxidant enzymes, including HO-1 and NAD(P)H quinone oxidoreductase-1 (NQO1), but inhibited Kelch-like ECH-associated protein 1 (Keap1) and NADPH oxidase type 4 (NOX4) (Ma et al. 2019). Finally, farrerol protected the retinal pigment epithelium cells from H_2_O_2_-associated oxidation by inhibiting ROS generation. Farrerol could ameliorate H_2_O_2_-induced cell death by activating Akt and MAPK and consequently increasing Nrf2/HO-1 generation in an adult retinal pigment epithelial cell line (Ma et al. 2021). Farrerol shows promise in treating or preventing age-related macular degeneration, acute liver injury, acute kidney injury, and oxidative stress-related diseases.

**Figure 2.**
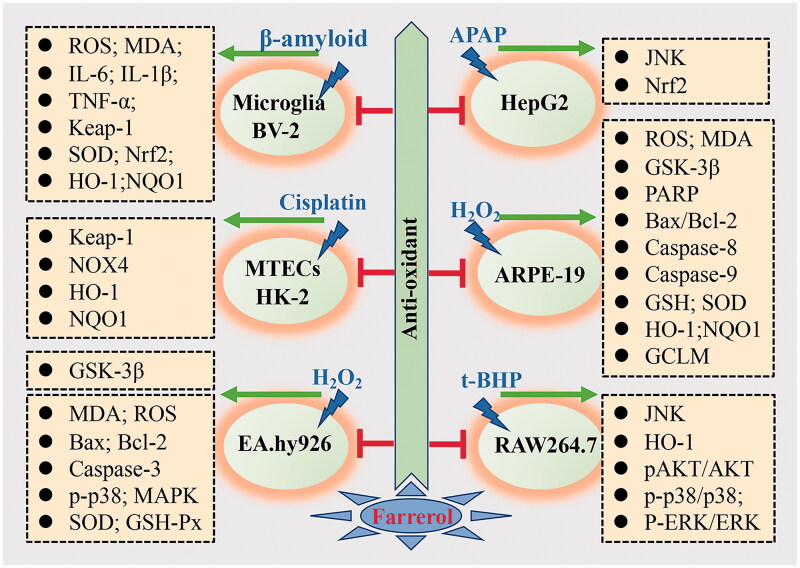
The anti-oxidant mechanism of farrerol and related molecules.

Nrf2 regulates the basal and inducible expression of antioxidant genes and other cytoprotective phase II detoxifying enzymes as key transcription factors (Eberhardt et al. 2012; Silva-Palacios et al. 2018; Chen Y et al. 2020). In addition, Cui, Zhang, et al. (2019) also revealed that the effect of farrerol on oxidative damage was mediated by the Nrf2/Keap1 signalling pathway. Chen et al. (2020) suggested that treatment with farrerol dose-dependently suppressed HG-induced mesangial cell damage through the Nox4/ROS/ERK/TGF-β signalling pathway. Besides, there is a new direction for the TGF-β1/Smad2 pathway to act as one of the downstream ERK1/2 pathways involved in farrerol-mediated anti-oxidative effects on HG-induced mesangial cell injury (Chen et al. 2020). Consistent with previous studies, our research team observed that farrerol exhibited protective effects on H_2_O_2_-induced EA.hy926 cells by enhancing superoxide dismutase (SOD) and glutathione peroxidase (GSH-Px) activities and inhibiting the elevation of intracellular MDA and ROS. In an ongoing study, farrerol could induce HO-1 and NQO1 expression, which are considered to be typical antioxidant enzymes against oxidative stress. The underlying mechanism is to specifically target GSK-3β and further activate the Nrf2-ARE signalling pathway (Yan et al. 2020). The sections are summarised in Table 1 and Figure 2.

Taken together, since HO-1, Nrf2, and NF-κB play a critical role in the antioxidant effect of farrerol, their specific and in-depth mechanisms require further investigation. Furthermore, farrerol may serve as a potential candidate for the treatment of cardiovascular diseases.

## Vasoactive effect of farrerol

Vascular diseases are the most common cause of death worldwide (Huynh 2017; Mencke et al. 2017). Commonly associated pathological mechanisms include endothelial dysfunction, vascular smooth muscle cell proliferation, and metal matrix protein deposition (Tousoulis et al. 2014; Uhrin et al. 2018; Carmo et al. 2019; Yang et al. 2019). Our team has conducted extensive work on the vasoactive mechanisms of farrerol and published eight papers. The main findings are as follows in Table 2.

**Table 2.** Vasoactive, antitumor and antimicrobial activity of farrerol and its related signalling pathway mechanism.

Activity	Cells/animal/bacteria	Mechanism	Signalling pathway	References
Vasoactive	VSMCs	↓α-SMA; SM22α; p-ERK/ERK; p-p38/p38	ERK1/2;	[Liu et al. 2020; Qin et al. 2019]
		**↑**OPN	p38 MAPK	
	VSMCs	LVGC	/	[Qin et al. 2014]
	VSMCs	Estrogen receptor-β	/	[Li et al. 2011]
	EA.hy926	↓p-ERK1/2	ERK1/2	[Li et al. 2014]
		**↑**Occludin; ZO-1		
	HUVECs; HMEC-1	↓the phosphorylation levels of ERK, Akt, mTOR, Jak2, Stat3; Bcl-2, Bcl-xl	Akt/mTOR; ERK; Jak2/Stat3	[Dai et al. 2016]
	SHR	↓NAD(P)H; p22^phox^	/	[Qin et al. 2015]
		**↑**eNOS		
	SHR	↓NAD(P)H	Vascular smooth muscle contraction	[Qin et al. 2018]
		**↑**VOC; ADRA1; AGTR1; CaM; IP_3_R; PLC		
Antitumor	SGC7901;	**↑**p27; ERK1/2; p38	ERK	[Liu et al. 2016]
	HUVECs			
	Calu-1	↓Slug; Zeb-1; Vimentin	/	[Li et al. 2018]
		**↑**E-Cadherin		
	SGC-7901	↓Bcl-2	Mitochondrial-mediated	[Liu et al. 2016]
		**↑**Caspase-3; Caspase-9；Bax		
Antimicrobial	*Staphylococcus aureus*	↓α-toxin	/	[Qiu et al. 2011]
	*Staphylococcus aureus*; Bovine mammary epithelial cells	↓Internalization of *S. aureus* into bMEC; NO production; NF-κB activation	/	[Yang et al. 2013]

In 2013, Li et al. (2013) evaluated the vasoactive effect of farrerol on the human endothelial EA.hy926 cells to prevent or treat for cardiovascular diseases, such as atherosclerosis, hypertension, and heart failure. The underlying mechanism may be related to the regulation of intracellular MDA and ROS levels; the expression of Bax, Bcl-2, cleaved caspase-3; and the phosphorylation of p38. Vascular endothelial permeability plays an important physiological role. Endothelial dysfunction is considered to be relevant to the pathogenesis of many cardiovascular diseases (Haybar et al. 2019). When there is a loss of membrane-associated adhesion molecules, vascular permeability increases excessively, adversely affecting blood vessels and organisms (Fang et al. 2018). Thus, in 2014, we investigated the effect of farrerol on the maintenance of vascular integrity. As part of our ongoing research, our results indicate that the regulation of occludin expression by farrerol in H_2_O_2_-induced EA.hy926 cells in a dose-dependent manner may be associated with the inhibition of ERK1/2 activation (Li et al. 2014).

Qin, et al. (2014) demonstrated the vasodilatory effect of farrerol in rat aortic vascular smooth muscle cells (VSMCs) for the first time; a possible mechanism is blocking Ca^2+^ release from the sarcoplasmic reticulum by the ryanodine receptors than by endothelium-derived vasodilator factors. Subsequently, in 2015, our research team reported that farrerol could attenuate the aortic lesions in spontaneously hypertensive rats (SHRs) by upregulating eNOS and decrease in NAD(P)H oxidase activity, which was also mediated by increased expression of eNOS and reduced p22^phox^ expression. Moreover, the results showed that farrerol partially reversed the morphological remodelling of the SHR aorta in media thickness, wall area, media-lumen ratio, and nuclei size (Qin et al. 2015). In 2017, our research team reported the discovery of ADRA1, a novel potential target gene for farrerol-treated SHRs through the gene expression profiling (Qin et al. 2018). In 2019, our team further verified the function of this gene in the contraction and relaxation of VSMCs. The experimental evidence suggested that farrerol could attenuate the rat aortic lesions, which involved inhibiting of the increased mRNA and protein expression of MLCK and SM22α and reducting of Ang II-induced increase in phosphorylation levels of MYPT1 and MLC by activating the α_1D_-adrenoceptor gene (Qin et al. 2019). In 2020, our research team indicated that farrerol could maintain the contractile phenotype of VSMCs partly by inactivating the ERK1/2 and p38 MAPK signalling pathways. Hence, we established that farrerol could prevent and treat vascular diseases as a natural product. At present, concerted efforts are being made to investigate the vasoactive effects of farrerol on vascular-related diseases.

In addition, we found that two other units studied the vasoactivity of farrerol. Li et al. (2011) showed that farrerol could inhibit FBS-induced VSMC proliferation as a functional phytoestrogen, which may be useful in preventing or treating cardiovascular diseases arising from abnormal VSMC proliferation. Li et al. (2016) suggested that farrerol could inhibit angiogenesis through the downregulation of the AKT/mTOR, ERK, and Jak2/Stat3 signalling pathways. In summary, we will provide new ideas by summarizing the reported studies on the vasoactivity of farrerol for further research.

## Antitumor effect of farrerol

Over the past few centuries, cancer incidence has continuously increased and become the primary cause of morbidity and mortality worldwide (Mi Ah et al. 2019; Wang et al. 2020). At present, the treatment modalities for cancer are divided into surgery, radiation therapy, and systemic treatment, including chemotherapy, targeted therapy, hormonal therapy, and immunotherapy (Miller et al. 2019; Ward et al. 2020). However, different therapies have their limitations and have severe adverse effect, such as chemotherapy-induced vascular toxicity (Ben-Aharon et al. 2012, 2015; Gupta et al. 2016). Farrerol has the potential to treat tumours.

Liu et al. (2016) observed selective cytotoxicity of farrerol against SGC7901 cells, but not HUVECs. Furthermore, their results showed that farrerol could inhibit cancer cell proliferation though G_0_/G_1_-phase cell cycle arrest mediated by sustained ERK activation. Furthermore, farrerol modulated the expression of EMT proteins to suppress the metastatic potential of lung squamous cell carcinoma (Li B et al. 2019), as shown in Table 2.

## Antibacterial effect of farrerol

The death toll due to antimicrobial resistance (AMR) may increase to 10 million globally by 2050, making the situation quite serious (Ghosh et al. 2019; Sanchez-Buso et al. 2021). Antibiotics have long been widely used and have good effects. However, the rapid changes in antimicrobial resistance have made it necessary for us to look for better options (Revie et al. 2018). Therefore, there is a pressing need to develop novel and potent antimicrobial agents to treat life-threatening infections.

Flavonoids, especially flavanones, have been considered as potential candidates for developing antibacterial and antifungal agents. Farrerol decreased the production of α-toxin by methicillin-sensitive *Staphylococcus aureus* and methicillin-resistant *S. aureus*. These experiments suggested that farrerol may inhibit the production of other exotoxin genes, including enterotoxins and toxic shock syndrome toxin 1, and enhance the expression of surface-related virulence factors (Qiu et al. 2011).

Previous studies demonstrated that farrerol (4–16 μg/mL) reduced the internalization of *S. aureus* into bMEC (Yang et al. 2013). Farrerol downregulated the mRNA expression of tracheal antimicrobial peptide (TAP) and bovine neutrophilb-defensin5 (BNBD5) in bovine mammary epithelial cells (bMECs) infected with *S. aureus*. In addition, farrerol treatment decreased nitric oxide (NO) production by bMECs after *S. aureus* stimulation. Farrerol suppressed *S. aureus*-induced NF-κB activation in bMECs but had no effect on bMEC viability. These results suggest that farrerol modulates the expression of TAP and BNBD5 gene in mammary glands, enhances bMECs defense against *S. aureus* infection and may be useful for protection against bovine mastitis.

In addition to its antibacterial effect, the antifungal effect of farrerol has also been investigated (Meragelman et al. 2005). Pharmacologists have previously demonstrated that farrerol can restrict the growth of certain important plant pathogenic fungi *in vitro* (Li et al. 2016). However, in a panel of 14 strains of *Candida*, farrerol was inactive against all test strains at the highest concentration tested (32 µg/mL) (Meragelman et al. 2005). Moreover, farrerol also showed moderate antibacterial activity against *B. cereus* (Li et al. 2016), as shown in Table 2.

## Conclusions

This review discusses the anti-inflammatory, antioxidant, vasoactive, antitumor, and antimicrobial activities of farrerol and its underlying molecular mechanisms, as shown by *in vitro* and *in vivo* studies.

Flavonoids, a major group, have been continuously explored and applied in therapeutics. In the last few decades, as we stated above, the pharmacological properties of farrerol have been constantly discovered, including anti-inflammatory, antioxidant, vasoactive, antitumor, antibacterial effects, etc. (Androutsopoulos et al. 2010; Maleki et al. 2019; Nagula and Wairkar 2019; Zeng et al. 2019). Their corresponding mechanisms are mainly focussed on the NF-κB, AKT, p38, ERK, and Nrf2-ARE signalling pathways. In 2020, a novel application of farrerol was first reported as a potentiator of CRISPR/Cas9-mediated genome editing through the high-throughput small-molecule screen identification (Zhang, Chen, et al. 2020). In this study, farrerol was shown to effectively facilitate precise targeted integration in human cells, mouse cells, and mouse embryos at multiple genomic loci. In addition, treatment of cells with farrerol did not have any obvious negative effects on genomic stability. Moreover, farrerol significantly improved the knock-in efficiency in blastocysts, and the subsequently generated knock-in mice retained the capacity for germline transmission.

Therefore, in view of its various characteristics, farrerol has great potential to address a wide range of current and future medical problems. Our review of the pharmacological activities and mechanisms associated with farrerol may provide new ideas for its use, especially in the context of the global COVID-19 epidemic, and help explore whether farrerol can enhance the immunity of the population against this disease (Riva et al. 2020; Meganck and Baric 2021). This review brings together the most recent studies in the field of farrerol and provides clues and basis for further research.
